# Poly(_L_-lysine)-*block*-poly(ethylene glycol)-*block*-poly(_L_-lysine) triblock copolymers for the preparation of flower micelles and their irreversible hydrogel formation

**DOI:** 10.1080/14686996.2024.2432856

**Published:** 2024-11-25

**Authors:** Yuta Koda, Yukio Nagasaki

**Affiliations:** a Department of Materials Science, Institute of Pure and Applied Sciences, University of Tsukuba, Tsukuba, Ibaraki, Japan; b Master’s School of Medical Sciences, Graduate School of Comprehensive Human Sciences, University of Tsukuba, Tsukuba, Ibaraki, Japan; c Center for Research in Radiation, Isotope and Earth System Sciences (CRiES), University of Tsukuba, Tsukuba, Ibaraki, Japan; d Department of Chemistry, Graduate School of Science, The University of Tokyo, Bunkyo-ku, Tokyo, Japan; e High-value Biomaterials Research and Commercialization Center (HBRCC), National Taipei University of Technology, Taipei, Taiwan

**Keywords:** Injectable hydrogels, polyion complex, sol-gel transition, poly(_L_-lysine)-*block*-PEG-*block*-poly(_L_-lysine) triblock copolymers, silica gel nanoparticles, modulus

## Abstract

Poly(_L_-lysine)-*block*-poly(ethylene glycol)-*block*-poly(_L_-lysine) (PLys-*block*-PEG-*block*-PLys) triblock copolymers formed polyion complex (PIC) with poly(acrylic acid) (PAAc) or sodium poly(styrenesulfonate) (PSS), leading to the formation of flower micelle-type nanoparticles (Nano^Lys/PAAc^ or Nano^Lys/PSS^) with tens of nanometers size in water at a polymer concentration of 10 mg/mL. The flower micelles exhibited irreversible temperature-driven sol-gel transitions at physiological ionic strength, even at low polymer concentrations such as 40 mg/mL, making them promising candidates for injectable hydrogel applications. Rheological studies showed that the chain length of PLys segments and the choice of polyanions significantly impacted irreversible hydrogel formation, with PSS being superior to PAAc for the formation. The incorporation of silica gel nanoparticles into the PIC flower micelles also resulted in irreversible gelation phenomena. The highest storage modulus exceeded 10 kPa after gelation, which is sufficient for practical applications. This study demonstrates the potential of these PIC-based hydrogels as biomaterials with tunable properties for biomedical applications.

## IMPACT STATEMENT

A polyion complex-based flower micelle, formed by poly(_L_-lysine)-*block*-PEG-*block*-poly(_L_-lysine) triblock copolymers and polyanions, shows irreversible hydrogel formation; incorporating silica enhanced its modulus to exceeding 10 kPa, promising for biomedical applications.

## GRAPHICAL ABSTRACT

**Figure:**
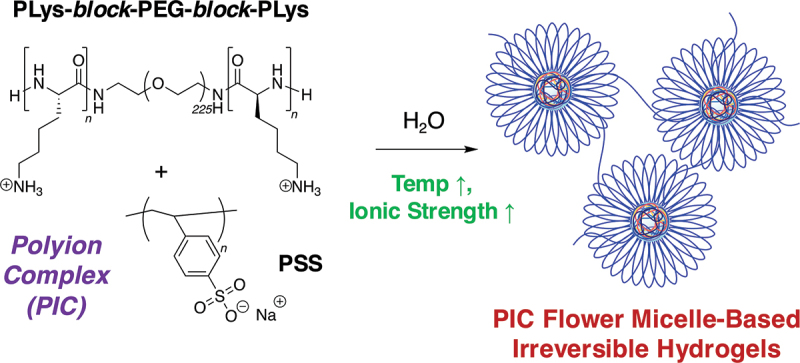

## Introduction

Injectable hydrogels are one of the attractive biomaterials administered into the body as liquids and converted to gels under certain conditions. These injectable gels are used for various applications such as tissue regeneration, local and sustainable drug delivery, orthopedics, regenerative medicine, and cosmetic surgery [1–5]. There are various types of injectable gels, such as ion-crosslinked gels like arginine, terminal reactive poly(ethylene glycols) (PEGs), and fibrin gels [6–8]. Recently, it has been reported that flower micelles were obtained using hydrophobic-hydrophilic-hydrophobic triblock copolymers, where the hydrophilic segment composed PEG, forming gels with increasing temperature [9–13]. These gels are in a one-liquid system, making them easy to handle because drugs can be incorporated into the flower micelles, and it is expected to be used in local and sustainable DDS [2,3,14–17]. Injectable gels must possess sol-gel transition properties allowing them to remain a liquid during injection and form a hydrogel with sufficient mechanical strength and viscosity under in vivo conditions. To achieve this, triblock copolymers such as poly(*ε*-caprolactone)-*block*-PEG-*block*-poly(*ε*-caprolactone) (PCL-*block*-PEG-*block*-PCL) were developed, and these copolymers form hydrogels upon heating [10,11,17–19]. In these systems, hydrophobic interactions of PEG shells, which increase with temperature, serve as driving force for hydrogel formation, leading to the aggregation of flower micelles [20,21]. A key challenge in injectable hydrogels formed by these flower micelles is that they often exhibit insufficient mechanical properties, such as low toughness and viscosity, once inside the body. For instance, hydrogels formed via hydrophobic interaction of PCL-*block*-PEG-*block*-PCL flower micelles typically have a modulus around only 10 Pa, which may not provide enough structural integrity to maintain their desired shape after injection. Another limitation of flower micelle-based hydrogels is the high concentration required for gelation. Typically, concentrations of 20 wt% or higher are necessary, which presents challenges for practical applications.

We recently reported that flower micelles, which use polyion complex (PIC) formation as core coagulation force, can form irreversible hydrogels even at low concentrations, and that these hydrogels exhibit higher mechanical strength than other flower-type micelles using hydrophobic core coagulation forces. For example, flower micelles are formed by combining a polystyrene-based triblock polymer (polyamine-*block*-PEG-*block*-polyamine) with a polyanion such as poly(acrylic acid) (PAAc), and PIC acts as coagulation force to form the micelle core [22–25]. Under the ionic strength of biological environment, these flower micelles undergo gelation with increasing temperature, achieving an elastic modulus exceeding 1 kPa. Interestingly, the hydrogel formation was irreversible and did not revert to a flowable sol even when the temperature was reduced. This suggests that PIC-driven flower micelle-type injectable gels hold potential for practical application. By incorporating antioxidant properties into this PIC-type injectable gel, we have developed a highly effective anti-tissue adhesion agent [24]. These PIC-type flower micelles and injectable gels can be also engineered as triblock copolymers with poly(amino acid) segments. For example, PIC flower micelles formed by mixing poly(arginine)-*block*-PEG-*block*-poly(arginine) with a polyanion successfully formed irreversible hydrogel within cardiac tissue when injected into a mouse model of myocardial infarction [26]. These hydrogels were degraded by inflammatory cells, such as macrophages, triggering a cascade of endogenous enzymatic reactions that convert poly(arginine) to arginine, and subsequently to nitric oxide, thereby promoting cardiovascular regeneration [26]. Thus, if injectable gels with sufficient mechanical strength can be developed using poly(amino acid)s, they could serve as promising matrices for local and sustained release of corresponding amino acids, thereby enhancing biological functions. However, the underlying mechanism and optimization of poly(amino acid)-based PIC flower micelles remain largely unexplored.

**Scheme 1.**
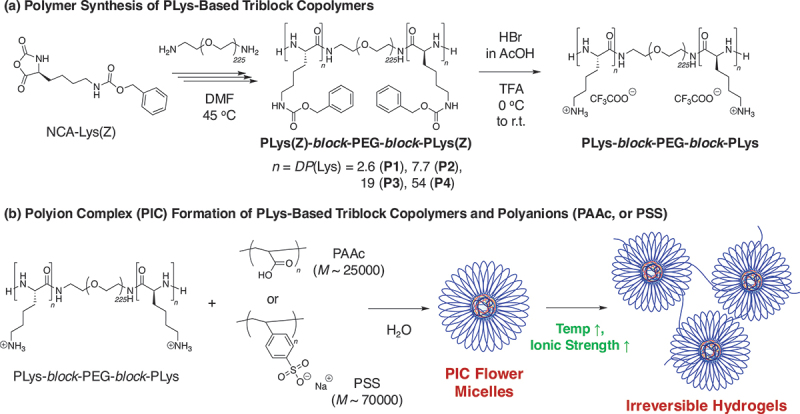
(a) Synthesis of poly(_L_-lysine) (PLys)-based triblock copolymers via ring-opening polymerization, and (b) design of polyion complex (PIC) flower micelle (Nano^Lys/PAAc^ or Nano^Lys/PSS^)-based hydrogels as injectable gels.

In this work, to deeply explore and optimize the performance of poly(amino acid)-based PIC micelles, we employed poly(_L_-lysine)-based triblock copolymers, poly(_L_-lysine)-*block*-PEG-*block*-poly(_L_-lysine) (PLys-*block*-PEG-*block*-PLys), with polyanions (Scheme 1). Herein, _L_-lysine (Lys) is one of the essential amino acids, and one of the attractive targeted amino acids because it has important biological functions [27–31]. If a bioavailable injectable gel based on PLys-*block*-PEG-*block*-PLys could be developed, it could be used for a variety of applications, such as local Lys release materials, combination with drug release, and wound dressings. It was confirmed that PLys-*block*-PEG-*block*-PLys formed PIC micelles in the aqueous condition in combination with PAAc or sodium poly(styrenesulfonate) (PSS). In these flower micelles, PEG formed the hydrophilic outer shells, while PIC formed the core structures, with the micelle size ranging from 10 to 46 nm. Under the micelle concentration over 40 mg/mL (~4 wt%), these flower micelles exhibited irreversible hydrogel formation driven by temperature at physiological ionic strength. In this study, we have focused on fundamental characterizations of sol-gel transitions of flower micelles, investigating the effects of ionic strength, chain length of PLys segments, and design of polyanions. In addition, we confirmed that when silica gel nanoparticles of 4 to 6 nm in size were mixed with flower micelles, they formed a complex in water and hydrogels with a higher elasticity by heating to 37°C under physiological salt concentrations. The resulting gels exhibited mechanical strength exceeding 10 kPa with initial micelle concentration of 40 mg/mL. Although almost all injectable gels with the high mechanical strength (~1 kPa) typically rely on two-liquid systems, we achieved high mechanical strength using a one-liquid system combined with silica gel nanoparticles. In fact, our silica-supported injectable gels exhibit a modulus approximately 10 times greater than that of the previous triblock copolymers. Injectable hydrogels, which are especially based on poly(amino acid) flower micelles formed by PIC interactions, exhibit great promise for a variety of biomedical applications due to their ability to form strong and irreversible gels at low concentrations ([polymer] = 40 mg/mL ~4 wt%). These injectable hydrogels will provide localized and sustained drug release, and promote tissue regeneration, making them highly potential candidates for practical use in fields such as cardiovascular therapy, drug delivery, and wound dressing.

## Materials and methods

### Chemicals

*N*^ε^-Carbobenzoxy-_L_-lysine (H-Lys(Z)-OH; TCI, purity > 98%), triphosgene (TCI, purity > 98.0%), (1*S*)-(-)-*α*-pinene (TCI, purity > 97.0%), HO-PEG-OH (Sigma-Aldrich, *M*_n_ ~10,000), methanesulfonyl chloride (MsCl; TCI, purity > 99.0%), triethylamine (Et_3_N; TCI, purity > 99.0%), chloroform (Fijifilm Wako Chemicals, purity ~99.0%), isopropanol (IPA; Fijifilm Wako Chemicals, purity ~ 98%), hexane (Fijifilm Wako Chemicals, purity ~95%), ammonia solution (Fijifilm Wako Chemicals, ~28 wt%), 1,2,3,4-tetrahydronaphtalene (tetralin; TCI, purity > 98.0%), a 33 wt% acetic acid solution of hydrogen bromide (HBr in AcOH; Sigma-Aldrich), trifluoroacetic acid (TFA; TCI, purity ~ 98.0%), poly(acrylic acid) (PAAc; Fijifilm Wako Chemicals, *M*_n_ ~25,000), and sodium poly(styrenesulfonate) (PSS; Sigma-Aldrich, *M*_w_ ~70,000) were used as received. Super dehydrated tetrahydrofuran (THF; Kanto Chemicals, purity > 99.5%) and *N,N*-dimethylformamide (DMF; Kanto Chemicals, purity > 99.5%) were further purified using a purification column (solvent dispensing system; GlassContour; Hansen & Co., Ltd.). The solutions of silica nanoparticles (Snowtex® XS, Snowtex® 30, Snowtex® XL, and Snowtex® ZL; Nissan Chemical Corporation) were also used as received. The corresponding nanoparticle diameters were 4–6 nm (Snowtex® XS), 10–15 nm (Snowtex® 30), 40–60 nm (Snowtex® XL), and 70–100 nm (Snowtex® ZL).

### Characterization of polymer materials

The number-averaged and weight-averaged molecular weights (*M*_n_ and *M*_w_, respectively) and the molecular weight distribution (*Đ*) of the polymers were measured using gel permeation chromatography (GPC) in DMF at 40°C (flow rate: 0.40 mL/min) on two polystyrene gel columns (TOSHO, Japan; TSKgel GMH_HR_-M; exclusion limit: molecular weight (MW) = 4.0 × 10^6^; particle size: 5 μm; pore size: N/A; 7.8 cm i.d. ×30 cm) connected to a pump (JASCO, Japan, PU-4180), refractive index (RI) detector (JASCO, Japan, RI-2031), and UV/Vis detector (JASCO, Japan, UV-4075). The columns were calibrated using 18 standard poly(ethylene oxide) (PEO) and poly(ethylene glycol) (PEG) samples (Merck; M_p_ = 238–1,180,000). ^1^H nuclear magnetic resonance (NMR) spectra were recorded in CDCl_3_ or DMSO-*d*_6_ at room temperature (22–23°C) using an AVANCE-600 NMR spectrometer (Bruker, USA) operating at 600 MHz (^1^H). Dynamic light scattering (DLS) measurements were conducted using Zetasizer Nano ZSP (Malvern, Netherlands) equipped with He – Ne laser (*λ* = 633 nm) at 37°C. The measurement angle was 173°, and the data were analyzed using nonnegative least squares (NNLS) method. Transmission electron microscopy (TEM) was conducted using a JEM-1400 (JEOL, Japan) at an accelerating voltage of 80 kV. The samples were prepared by drop-casting aqueous solutions of the flower micelle-type nanoparticles ([polymers] = 10 mg/mL) onto carbon-coated grids (OKENSHOJI, Japan; ELS-C10 STEM Cu100P) and staining with a 1 wt% phosphotungstic acid solution (10 μL, pH = 7.4).

### Synthesis of MsO-PEG-OMs

HO-PEG-OH (27.2 g, 2.72 mmol; 1.0 eq.) was placed into a 300 mL round-bottomed flask. Chloroform (70 mL), Et_3_N (3.8 mL, 27 mmol; 10 eq.), and MsCl (2.1 mL, 27 mmol; 10 eq.) were added to the flask, and the solution was stirred at room temperature for 5 h. The product was precipitated by cold IPA (−30°C) and collected by centrifugation (−10°C, 9000 rpm, 10 min). The obtained product was then solubilized by methanol and the precipitation process was repeated three more times. Finally, a white product was obtained and dried under reduced pressure (26.8 g, Yield 99%).

### Synthesis of H_2_N-PEG-NH_2_

MsO-PEG-OMs (54.3 g, 5.43 mmol) was placed into a 2000 mL round-bottomed flask, and an aqueous NH_3_ solution (380 mL) was poured into the flask. After solubilization of MsO-PEG-OMs at 50°C, the solution was stirred at room temperature for 4 days. The solvent was removed by a vacuum pump, and the obtained product was solubilized by methanol. The precipitation using IPA, which was same as the purification of MsO-PEG-OMs, was conducted four times. A white product was finally obtained and dried under reduced pressure (53.6 g, Yield 99%).

### Synthesis of α-amino acid N-carboxyanhydride of Lys(Z) (NCA-Lys(Z)) monomer

H-Lys(Z)-OH (25.2 g, 89.9 mmol; 1.0 eq.) and triphosgene (18.2 g, 61.3 mmol; ~ 0.67 eq.) were placed into a 1000 mL round-bottomed flask. *α*-Pinene (36 mL, 227 mmol; 2.5 eq.) and THF (252 mL) were poured to the flask, and the heterogeneous mixture was stirred at 50°C for 3 h. After cooling to room temperature, the homogenous solution was dropped into hexane and a precipitate was obtained. The precipitate was then washed by hexane several times. After storage of the hexane solution at −30°C overnight, the product was collected by filtration. The product was then solubilized by mixed solvent of THF/acetone/IPA (~10/10/1, v/v/v) again, and the precipitation process was repeated two more times. Finally, a white powder was obtained and dried under reduced pressure (16.4 g, Yield 59%).

### Synthesis of poly(_L_-lysine(Z))-based triblock copolymers

PLys(Z)-*block*-PEG-*block*-PLys(Z) triblock copolymers (**P1** – **P4**) were synthesized using a syringe technique under N_2_ in a round bottomed flask with a three-way stopcock via ring-opening polymerization. The typical procedure is described below.

**P2**: H_2_N-PEG-NH_2_ (25.1 g, 2.51 mmol) was placed into a 500 mL round bottomed flask. The initiator was dried using a vacuum pump for 30 min, and the flask was purged by N_2_. DMF (44.8 mL), which was dried over activated molecular sieves 4A (MS4A), was added to the flask under N_2_, and the initiator was completely solubilized. Tetralin (0.3 mL) and a 1000 mM  DMF solution of NCA-Lys(Z) (70.4 mL, 70.4 mmol) were sequentially added to the flask in this order. The solution was stirred at 45°C for 4 days, and the reaction was quenched by liq. N_2_. The conversion of NCA-Lys(Z) was determined by ^1^H NMR using tetralin as internal standard. The product was precipitated by hexane/cold IPA (= 1/1, v/v), and the obtained product was collected by centrifugation (−10°C, 9000 rpm, 10 min). The obtained product was then solubilized by DMF and the precipitation process was repeated two more times. The product after precipitation was dried under reduced pressure (30.5 g). Other triblock copolymers (**P1**, **P3**, and **P4**) were synthesized in the same manner.

### Deprotection of Z groups on PLys(Z) segments

PLys(Z)-*block*-PEG-*block*-PLys(Z) triblock copolymer (**P2**; 30.5 g, 33.6 mmol (Lys(Z)); 1.0 eq.) was placed into a 500 mL round-bottomed flask, and TFA (300 mL) was poured to the flask. The solution was cooled using an ice bath, and an AcOH solution of HBr (61 mL, 337 mmol; 10 eq.) was slowly added. The solution was stirred with an ice bath overnight. The reaction was quenched by saturated aqueous solution of Na_2_CO_3_. The solution was dialyzed against MeOH/aq. Na_2_CO_3_ (~1/1, v/v) for 3 days, and against water for 4 days (Spectra/Snowtex® 3; molecular weight cut-off (MWCO) 3500). The inner solution was lyophilized, and PLys-*block*-PEG-*block*-PLys triblock copolymer (**P2**) was obtained (19.6 g). Z groups in other triblock copolymers (**P1**, **P3**, and **P4**) were deprotected in the same manner.

### Preparation of the polyion complex (PIC) micelle solutions

PLys-*block*-PEG-*block*-PLys triblock copolymers were solubilized by DMSO/TFA (= 20/1, v/v; [polymer] = 5 mg/mL). Polyanions (PAAc or PSS) were solubilized by phosphate-buffered (PB) saline ([polymer] = 5 mg/mL), and each PB solution of polyanions was slowly added to the solution of PLys-*block*-PEG-*block*-PLys triblock copolymers. The mixture was dialyzed against water (Spectra/Por® 3; MWCO 3500). The inner aqueous solutions of PIC micelles (Nano^Lys/PAAc^ or Nano^Lys/PSS^) were condensed using a centrifugal evaporator (EYELA, CVE-3100). The concentration of the resulting solution was determined by weighing the obtained polymer after lyophilizing 100 μL of the solution.

### Viscoelastic properties characterized using a rheometer

The viscoelastic properties of PIC micelles were characterized using a rheometer (MCR302, Anton Paar) in the same way as the previous work [20]. In this work, we have measured shear storage and loss moduli (*G’* and *G”*) and complex viscosity ([η*]) for the characterization of gelation process and hydrogel properties. The storage and loss moduli indicated solid and liquid properties, respectively. For example, a material mainly shows solid properties when the storage modulus is higher than loss modulus (*G’* > *G”*). Furthermore, the gelation point can be determined at the cross-point where both moduli show the same values (*G’* = *G”*). In this work, a parallel plate with 20 mm diameter and a gap of 0.2 mm was used. The viscoelastic properties were monitored by changing the temperature from 15°C to 45°C at a fixed frequency of 1 Hz, and the heating and cooling rate was set as 1°C/min. Each aqueous solution of silica gel nanoparticles, which was the suspension solution, was added to the aqueous solution of PIC micelles (Nano^Lys/PAAc^ or Nano^Lys/PSS^) right before the characterization using a rheometer, and the solutions were mixed well by a vortex for 3 min.

## Results and discussion

### Polymer synthesis

Amphiphilic ABA-type triblock copolymers such as poly(*ε*-caprolactone)-*block*-PEG-*block*-poly(*ε*-caprolactone) form flower-type micelles under aqueous condition. PEG chains in these flower micelles increase hydrophobicity by heating, and the flower micelles aggregate with each other. The entropy of PEG chains of these flower micelles is reduced because both chain ends of PEG on the surface are anchored to the core, making PEG more sensitive to temperature changes. As the temperature rises, hydration of PEG chains decreases, causing them to become more hydrophobic. The flower micelles aggregate, leading to the formation of bridging [20,21]. This is the mechanism of the hydrogel formation using flower micelles composed of amphiphilic triblock copolymers, where PEG serves as hydrophilic polymer segment. Since this mechanism relies solely on the aggregation of micelles caused by hydrophobization of PEG induced by heating, these hydrogels do not exhibit high elastic moduli, and the moduli are often around 10 Pa.

**Figure 1.**
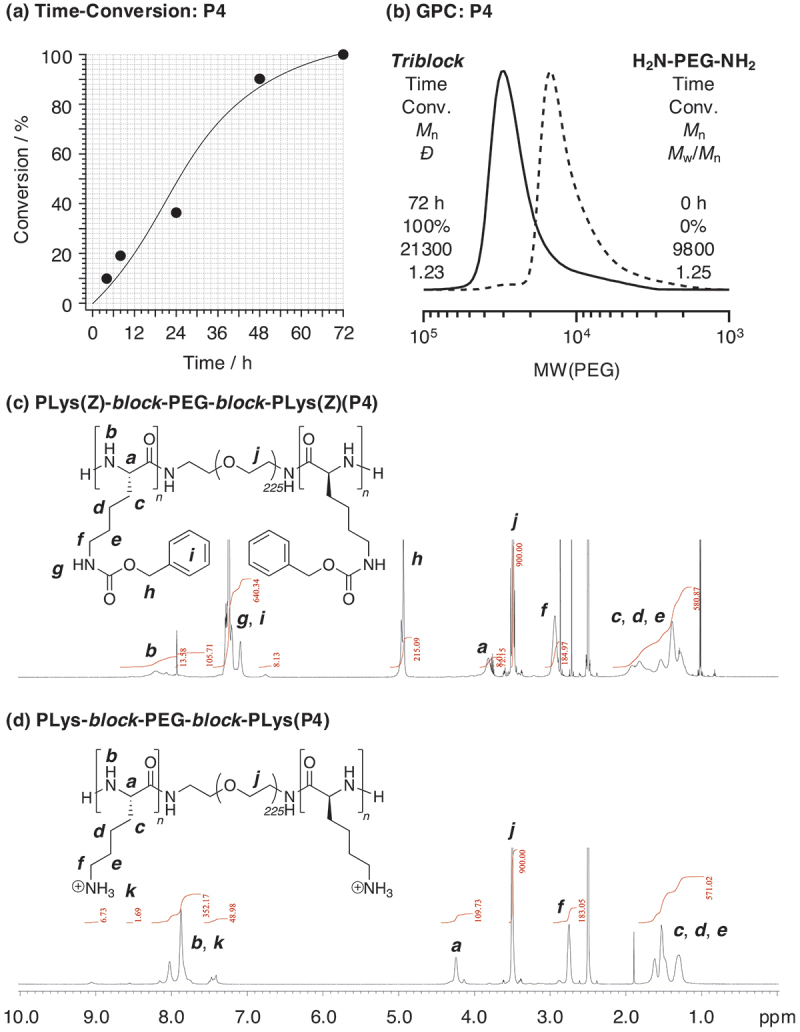
(a) Time-conversion of NCA-Lys(Z) and (b) GPC curves for synthesizing PLys(Z)-*block*-peg-*block*-PLys(Z)(P4) triblock copolymers in DMF at 45°C (condition: [NCA-Lys(Z)]/[H_2_N-PEG-NH_2_] = 1000/9.0 mM). ^1^H NMR spectra (600 MHz) of (c) PLys(Z)-*block*-peg-*block*-PLys(Z)(P4), and (d) PLys-*block*-peg-*block*-PLys(P4) in DMSO-*d*_6_/TFA = 20/1 (v/v) at 25°C ([polymer] = 5 mg/mL, *δ* = 2.50 ppm (DMSO)).

As explained above, in PIC flower micelles that form on ionic interactions, the gelation is influenced not only by the aggregation resulting from hydrophobization of PEG upon heating but also by changes in coagulation forces within the PIC. As the ionic strength increases, the PIC core loosens due to electrostatic shielding effects, causing some polymer chains to be released and exposed from the core. These exposed chains form bridge structure, leading irreversible hydrogel formation. These factors are key contributors to the gelation process in PIC-driven flower micelles (Scheme 1). Here, we employed triblock copolymers based on poly(amino acid) as polycations to investigate the formation and gelation behavior of flower micelles driven by polyion complexes. In this work, we used PLys-*block*-PEG-*block*-PLys as a polycationic copolymer prepared via ring-opening polymerization of NCA-Lys(Z) using a H_2_N-PEG-NH_2_ macroinitiator in DMF at 45°C [32–34]. To investigate the effect of the number of cations on the polycationic segments on the gel formation properties, triblock copolymers with different degrees of polymerization (DP) of Lys units in a PLys segment (*DP*(Lys)) were prepared. The targeted DP was varied from 5 to 50 (*DP*(Lys)_target_ = 5(**P1**), 10(**P2**), 20(**P3**), and 50(**P4**)). In all cases, NCA-Lys(Z) was smoothly consumed, and PLys(Z)-*block*-PEG-*block*-PLys(Z) triblock copolymers were obtained (Table 1; Figure 1a,b; Figure S1; *M*_n_(GPC) = 10200–21300, *M*_w_(GPC) = 12000–26000, *Đ*(GPC) = 1.07–1.23). All signals in ^1^H NMR spectrum of **P4** are assigned to PLys(Z)-*block*-PEG-*block*-PLys (Z), suggesting successful synthesis of desired triblock copolymers (Figure 1c). Other results are listed in Table 1, and their GPC results are shown in the supplementary information (Figure S1). *M*_n_ and *DP*(Lys) were determined by the peak ratio between PEG and benzyl groups, and *M*_n_(NMR) = 11400–38100 and *DP*(Lys)_NMR_ = 2.7–54 (Table 1, Figure 1c).

**Table 1.** Synthesis of Synthesis of Poly(_L_-lysine)-Based Triblock Copolymers.

Code	Side Chains	*DP*(Lys)_target_^*a*^	*M*_n_^*c*^ (GPC)	*Đ*^*c*^ (GPC)	*DP*(Lys)_NMR_^*d*^	*M*_n_^*d*^ (NMR)	*D*_H,volime_(PAAc)^*e*^ /nm	*D*_H,volime_(PSS)^*f*^ /nm
P1	Z	5	10200	1.18	2.7	11400	–	–
	None		–	–		10700	40	10
P2	Z	10	15300	1.07	7.7	14100	–	–
	None		–	–		12000	19	15
P3	Z	20	17000	1.07	19	20100	–	–
	None		–	–		15000	20	25
P4	Z	50	21300	1.23	54	38100	–	–
	None		–	–		23700	46	43

^*a*^Targeted degree of polymerization (DP) at 90% monomer conversion: *DP*(Lys)_target_ = 0.90 × [NCA-Lys(Z)]/[H_2_N-PEG-NH_2_]. Polymerization condition: [NCA-Lys(Z)]/[H_2_N-PEG-NH_2_] = (**P1**) 500/45, (**P2**) 500/18, (**P3**) 500/9, and (**P4**) 1000/9 mM in DMF at 45°C. ^*c*^ Number-average molecular weight (*M*_n_) and distribution (*Đ*) determined by gel permeation chromatography (GPC) in DMF ([LiBr] = 10 mM) with poly(ethylene oxide) (PEO) standards. ^*d*^ The observed *DP* (*DP*(Lys)_NMR_) was determined using PLys(Z)-*block*-PEG-*block*-PLys(Z) by ^1^H NMR in DMSO-*d*_6_/TFA = 20/1 (v/v) ([polymer] = 5 mg/mL). The number-average molecular weight (*M*_n_(NMR)) was determined by the observed *DP*(Lys)_NMR_. ^*e*^ The volume-average hydrodynamic diameter (*D*_H,volume_) of PIC micelles with PAAc were determined by DLS in water at 25°C ([polymer] = 10 mg/mL). ^*f*^ The volume-average hydrodynamic diameter (*D*_H,volume_) of PIC micelles with PSS were determined by DLS in water at 25°C ([polymer] = 10 mg/mL).

The deprotection of Z groups in PLys(Z) segments was carried out using HBr in TFA to produce triblock-based polycations (PLys-*block*-PEG-*block*-PLys) with a free amino group as a side chain of each Lys repeating unit. The peak from benzyl protons (peak ***h***) in ^1^H NMR spectra before deprotection completely disappeared after the reaction (Figure 1d). These results supported successful synthesis of PLys-*block*-PEG-*block*-PLys (Table 1, Figure 1d; *M*_n_(NMR) = 10700–23700).

### Preparation of flower micelles

**Figure 2.**
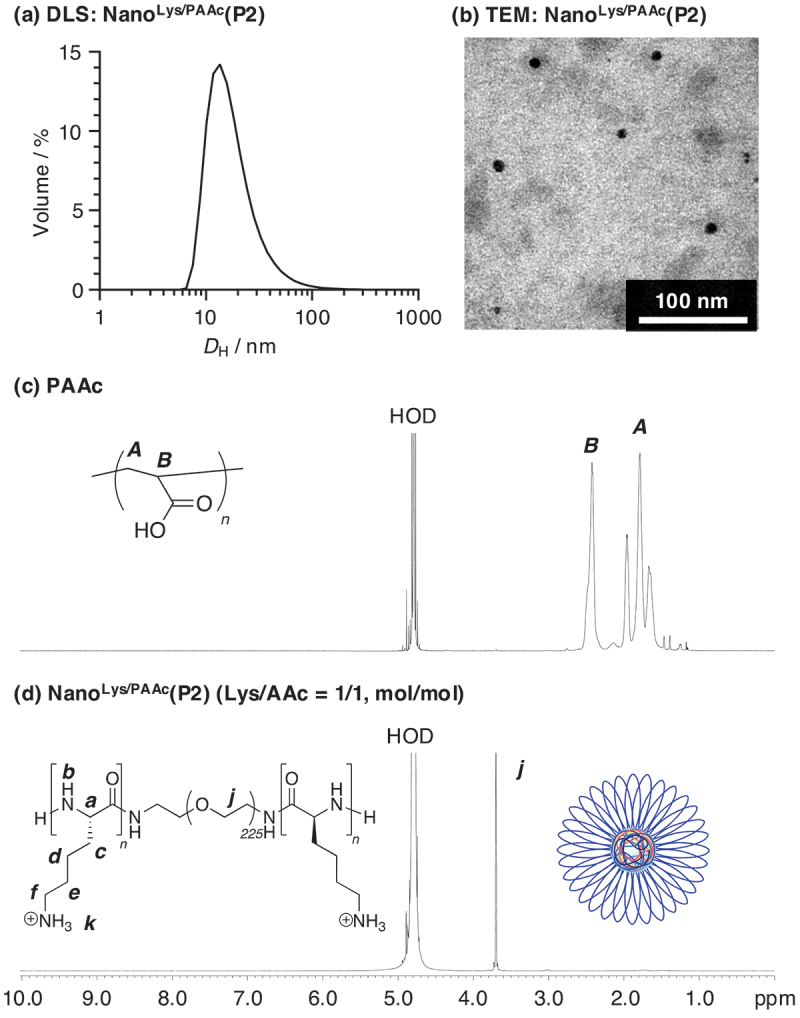
(a) Volume distribution of hydrodynamic diameter and (b) a TEM image of Nano^Lys/PAAc^(P2) in water at 25°C ([polymer] = 10 mg/mL, lys unit in PLys-*block*-peg-*block*-PLys(P2)/AAc unit in poly (acrylic acid) (PAAc) = 1/1 (mol/mol)). ^1^H NMR spectra (600 MHz) of (c) PAAc and (d) Nano^Lys/PAAc^(P2) ([polymer] = 5.0 mg/mL, *δ* = 4.79 ppm (HOD)).

Mixing an equal amount of oppositely charged polyion pairs generates water-incompatible regions, leading to reduction in system entropy [35]. However, this decrease in entropy is compensated by release of counterions from both charged segments, resulting in formation of a highly stable polyion complex (PIC). Thus, the formation of flower micelles driven by PIC force is anticipated when the aforementioned PLys-*block*-PEG-*block*-PLys triblock copolymers are combined with polyanions. PAAc and PSS were utilized as polyanions in this work, and each PB solution of PAAc or PSS was added to each DMSO solution of PLys-*block*-PEG-*block*-PLys triblock copolymers (**P1**–**P4**). After dialysis against water, the solutions were characterized by DLS at 25°C ([polymer]_total_ = 10 mg/mL; Lys unit in PLys-*block*-PEG-*block*-PLys/AAc or SS unit in polyanions = 1/1 (mol/mol)). Both PAAc and PSS afforded nanoparticles (Nano^Lys/PAAc^ or Nano^Lys/PSS^) with **P1**–**P4**, and the volume distributions of the hydrodynamic diameter (*D*_H,volume_) were 10–46 nm (Table 1, Figure 2, Figure S2–S4).

For further investigation, PIC formation between PAAc and PLys-*block*-PEG-*block*-PLys(**P2**) was characterized by ^1^H NMR in D_2_O (Figure 2c,d; [polymer]_total_ = 5 mg/mL). While PAAc exhibited proton signals corresponding to their environments in D_2_O (Figure 2c), only signals corresponding to PEG segments were observed in the spectrum of PLys-*block*-PEG-*block*-PLys/PAAc mixture (Figure 2d). This indicates that both polyion chains are embedded into the solid core of flower micelles (Nano^Lys/PAAc^(**P2**)), with PEG forming hydrophilic shells. The absence of polyion signals in the ^1^H NMR spectrum can be attributed to the significantly shortened relaxation times of the segments within the solid core, resulting from their restricted mobility, thereby rendering the peaks undetectable. PSS and PLys-*block*-PEG-*block*-PLys(**P2**) was also characterized by ^1^H NMR in D_2_O, and same results were obtained (Figure S4), indicating that PSS also afforded PIC-based flower micelles (Nano^Lys/PSS^(**P2**)) together with PLys-*block*-PEG-*block*-PLys(**P2**).

### Rheological analysis of poly(lysine)-based PIC flower micelles

**Figure 3.**
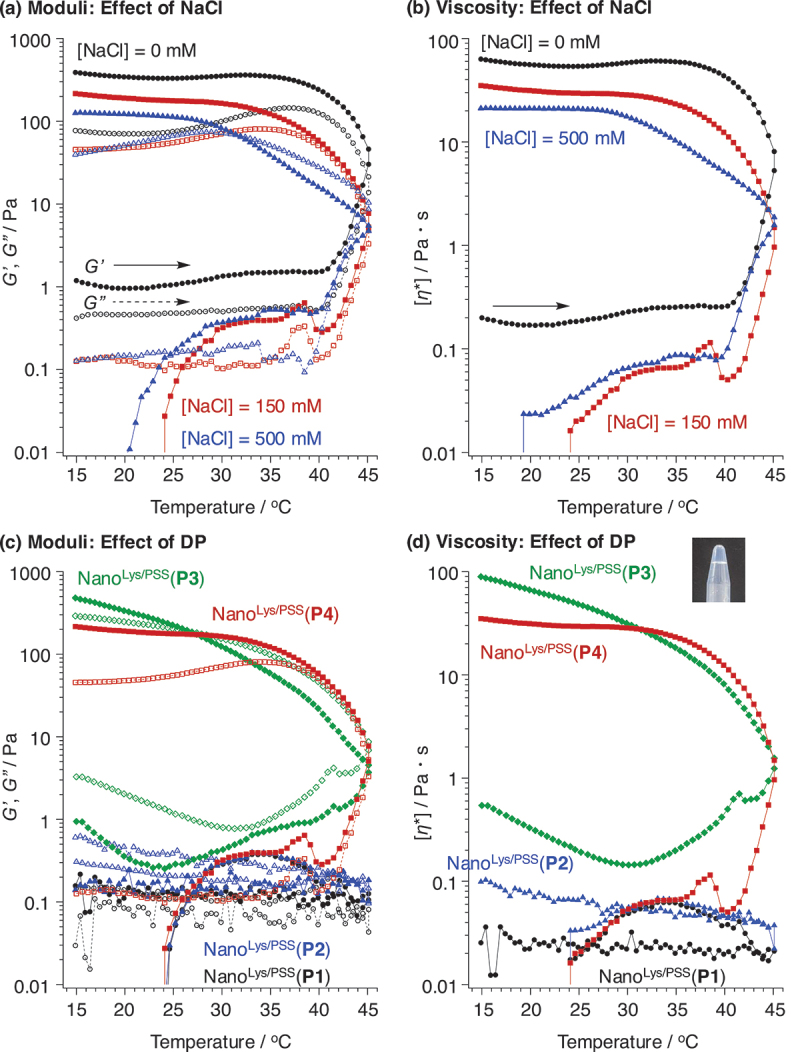
Effects of (a, b) the ionic strength and (c, d) *DP*(Lys) on (a, c) the storage (*G’*; filled marks, solid lines) and loss (*G”*; opened marks, dashed lines) moduli, and (b, d) the complex viscosity of Nano^Lys/PSS^(a and b, P4; c and d, P1–P4) ([polymers] = 40 mg/mL in water; monomer unit ratio, Lys unit in PLys-*block*-peg-*block*-PLys/styrenesulfonate unit in sodium poly(styrenesulfonate) (PSS) = 1/1 (mol/mol), heating and cooling rate = 1 ℃/min). Condition: (a, b) [NaCl] = (black circles) 0, (red squares) 150, and (blue triangles) 500 mM. (c, d) [NaCl] = 150 mM, and (black circles) P1, (blue triangles) P2, (green rhombus) P3, and (red squares) P4. The picture in (d) showed the irreversible gelation of Nano^Lys/PSS^(P3) after cooling to room temperature.

In developing injectable hydrogels for biomedical applications, low viscosity around room temperature, and irreversible and rapid gelation at around body temperature are desirable. It is also crucial to ensure that the gel maintains sufficient mechanical strength throughout the required period after gelation. To investigate the fundamental characteristics of flower micelles prepared in this study, rheological analysis was performed by measuring the shear storage modulus, loss modulus (*G’* and *G”*), and complex viscosity ([η*]) of PIC flower micelles as a function of temperature. As described above, the driving force for PIC formation is the decrease in system entropy when polycations and polyanions interact with each other, and this decrease in entropy is compensated by the release of counterions of both polycation and polyanion chains [35]. After the PIC formation, the ionic strength of the solution is one of the important factors for the stability of PIC. The formed PIC becomes more loosened in accordance with the increase in the concentration of ionic species. As ionic strength rises, the electrostatic interaction between the polycation and polyanion is shielded, weakening the PIC core coagulation force. Consequently, some polymer chains are exposed and released from the PIC core, and some of them act as bridge chains between flower micelles and others exist as isolated chains [20,21]. This is the mechanism of irreversible hydrogel formation. Therefore, it is essential to quantitatively assess the impact of ionic strength on the stability of these PIC flower micelles. Thus, we also investigated the effect of ionic strength on hydrogel formation using Nano^Lys/PSS^(**P4**) by increasing the concentration of NaCl (Figure 3a,b; [polymer] = 40 mg/mL; [Lys unit] = 67 mM). Herein, the concentration of Lys units in triblock copolymers was 67 mM. Therefore, the ionic species significantly interfered with PIC core structure in the presence of a 150 mM NaCl solution. Although the storage modulus was higher than loss modulus in the absence of NaCl, the viscosity of Nano^Lys/PSS^(**P4**) was low below 40°C (~0.2 Pa s). This means that Nano^Lys/PSS^(**P4**) without electrolytes was injectable. During heating, gelation was confirmed by the fact that the viscosity immediately increased in accordance with increase of storage modulus, and gelation points were respectively 25.9 and 24.7°C under the condition of 150 and 500 mM  NaCl solutions (Table S1; heating rate = 1°C/min). The gelation temperature decreased by increasing the concentration of NaCl, therefore, this gelation was driven by both temperature and ionic strength. One of the important points of this gelation was the irreversible hydrogel formation, i.e. the storage modulus did not revert to the initial values during the cooling process (cooling rate = 1°C/min) and kept high values. Another key factor in the fundamental characterization of our hydrogels is that the preparation process, including the time-course of temperature change may influence both the irreversible hydrogel formation and its properties. Additionally, the significant step change in storage modulus observed between 25–30°C and 40–45°C suggests molecular level changes. We will continue to investigate the effects of the preparation process on the hydrogels as well as the intermediate structures, in detail.

To clear the effect of *DP*(Lys) on irreversible hydrogel formation under physiological condition ([NaCl] = 150 mM), all Nano^Lys/PSS^(**P1**–**P4**)s were similarly characterized by a rheometer as above (Figure 3c,d). Nano^Lys/PSS^(**P1**) and Nano^Lys/PSS^(**P2**) did not form hydrogels, confirmed by their low viscosity (<0.1 Pa s). This might be because their coagulation forces in PLys chains were not sufficient to form a stable PIC-core under physiological conditions. In the case of Nano^Lys/PSS^(**P3**) and Nano^Lys/PSS^(**P4**), both their storage moduli and viscosity increased with temperature, leading to irreversible hydrogel formation. This indicates that PLys segments require more than ~20 units of Lys for irreversible formation under the present concentration conditions. Since the hydrogel formation process is irreversible, the preparation conditions, including the rate of temperature change, might influenced the irreversible gelation as well as the properties, and we will continue to investigate the effect of preparation conditions on the hydrogel formation and properties.

**Figure 4.**
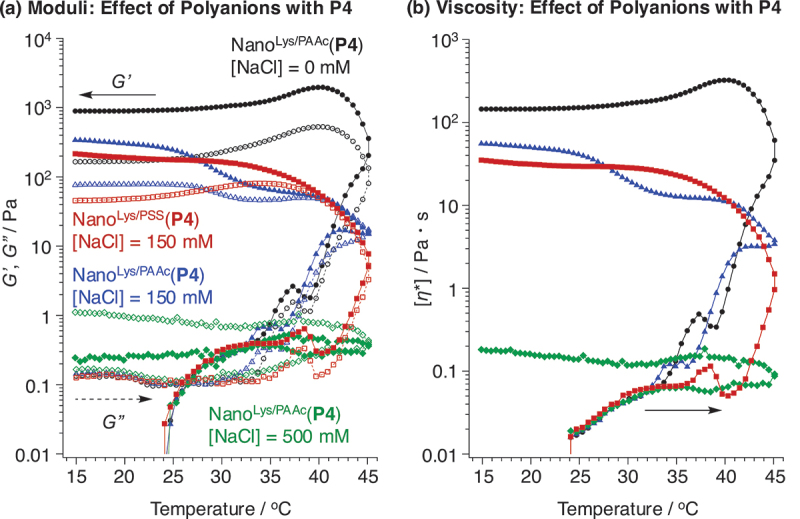
Effects of (a, b) the polyanions and (c, d) the acylated amphiphilic copolymers on (a, c) the storage (*G’*; filled marks, solid lines) and loss (*G”*; opened marks, dashed lines) moduli, and (b, d) the complex viscosity of Nano^Lys/Polyanion^-based hydrogels (PAAc or PSS) ([polymers] = 40 mg/mL in water; monomer unit ratio, lys unit in PLys-*block*-peg-*block*-PLys(P4)/anion unit in polyanions = 1/1 (mol/mol); heating and cooling rate = 1°C/min). Condition: [NaCl] = (black circles) 0, (red squares, blue triangles) 150, and (green rhombus) 500 mM.

The effect of polyanion on the irreversible hydrogel formation was investigated using Nano^Lys/PSS^(**P4**) and Nano^Lys/PAAc^(**P4**) (Figure 4). In the case of 0 and 150 mM  NaCl solutions, Nano^Lys/PAAc^(**P4**) also showed irreversible hydrogel formation, and its storage moduli and viscosity increased with temperature. However, Nano^Lys/PAAc^(**P4**) did not induce gelation in the case of 500 mM NaCl solution, as indicated by the fact that the loss modulus was exceeding storage modulus again in addition to low viscosity. This phenomenon is likely due to the weaker acidity of PAAc than PSS (p*K*_a_ = 6.21 (PAAc) and 1.22 (PSS)) [36]. This unique gelation phenomenon, driven by changes in ionic strength, has potential applications such as: when the PIC micelle solution is delivered to a target site via a catheter, a 500 mM solution is anticipated to remain fluid within the catheter and only form gels once released into the body, where the ionic strength is lower. Another point was that the gelation temperature did not depend on the concentration of NaCl, and the gelation temperature during the heating process was approximately 27°C in all cases, regardless of ionic strength. The exact reason for this remains unclear, but it is believed that gelation of Nano^Lys/PAAc^ with increasing temperature is primarily triggered by micelle aggregation due to hydrophobization of PEG chains, rather than by core loosening caused by changes in ionic strength. It is also important to note that the molecular weights of polyanions were not identical in this study. We will continue to investigate the effect of polyanion molecular weight on the hydrogel formation and properties in more detail.

### Enhancement of mechanical strength using silica gel nanoparticles

**Figure 5.**
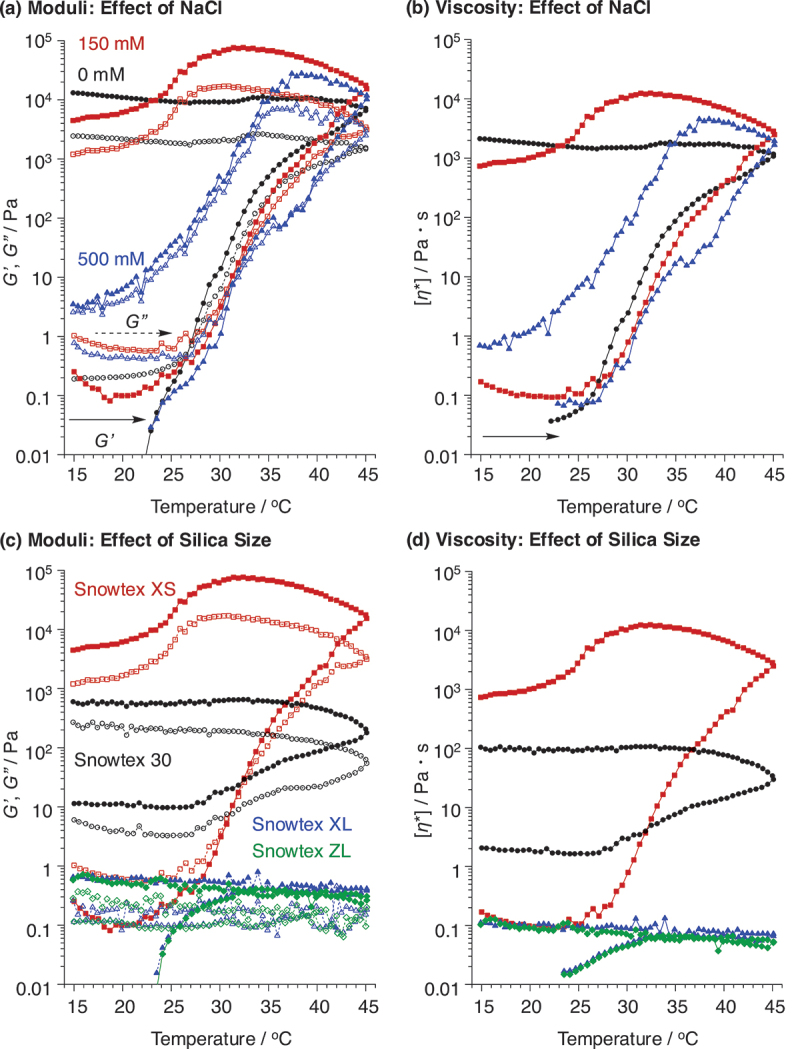
Effects of the size of silica gel nanoparticles on (a) the storage (*G’*; filled marks, solid lines) and loss (*G”*; opened marks, dashed lines) moduli, and (b) the complex viscosity of Nano^Lys/PSS/SiO^(P4)([polymers] = 40 mg/mL in water; monomer unit ratio, lys unit in PLys-*block*-peg-*block*-PLys(P4)/styrenesulfonate unit in PSS = 1/1 (mol/mol); [NaCl] = (a and b, black circle) 0, (a and b, red square; c and d) 150, and (a and b, blue triangle) 500 mM, [silica gel nanoparticles] = 56 mg/mL; heating and cooling rate = 1 ℃/min). (c, d) the nanoparticle sizes were (Snowtex® XS; red squares) 4–6 nm, (Snowtex® 30, black circle) 10–15 nm, (Snowtex® XL, blue triangles) 40–60 nm, and (Snowtex® ZL, green rhombus) 70–100 nm.

While PLys-based triblock copolymers formed PIC micelles with polyanions and exhibited irreversible hydrogel formation, the storage modulus after gelation in all conditions was less than 1 kPa. To enable versatile applications, it is preferable to increase the storage modulus by at least 1 kPa. To investigate the potential for enhancing mechanical strength during the gelation process, we incorporated silica gel nanoparticles with negatively charged surfaces into Nano^Lys/PSS^(**P4**). Even though enhancement of mechanical strength is not achieved, silica gel nanoparticles show great potential as drug carriers, with clinical trials already underway in the United States. Therefore, combining silica gel nanoparticles with flower micelles that possess injectable gel properties could significantly expand their potential applications. In this work, we have used four silica gel nanoparticles (Snowtex®) to study the effect of combination of silica nanoparticles with different sizes of 4–6 nm (Snowtex® XS; ST-XS), 10–15 nm (Snowtex® 30; ST-30), 40–60 nm (Snowtex® XL; ST-XL), and 70–100 nm (Snowtex® ZL; ST-ZL). After incorporation of Snowtex® XS into Nano^Lys/PSS^(**P4**), its size increased to 632 nm, indicating an interaction between Snowtex® XS and Nano^Lys/PSS^(**P4**), with no precipitation of the composite nanoparticles observed during DLS measurements (Figure S5; [polymer] = 1.0 mg/mL). Other silica nanoparticles exhibited a similar tendency toward Snowtex® XS, with the size of the resulting aggregates ranging from 88 to 1117 nm (Figure S5 and S6). Although DLS analyses suggested interaction between flower micelles and silica gel nanoparticles, the internal structure of the composite nanoparticles remain unclear, requiring further investigation. Interestingly, the storage modulus of the hybrids increased significantly with rising temperature. For example, the composite of Nano^Lys/PSS^ with Snowtex® XS (Nano^Lys/PSS/SiO^(**P4**;ST-XS)) exhibited gelation as the temperature increased, achieving a storage modulus exceeding 10 kPa, even at such an initial silica concentration of just 56 mg/mL (Figure 5a,b). The storage modulus after gelation was further increased in the presence of NaCl. This might be due to the partial disintegration of PIC-core induced by electrostatic shielding effects, leading to the exposure of its dangling PLys chains outside the core and formation of bridge chains with added silica gel nanoparticles as illustrated in Figure S7. The formation of these bridge chains between flower micelles and silica gel nanoparticles might increase the mechanical strength of the gels. The gelation temperature was unaffected by the NaCl concentration in the presence of silica gel nanoparticles, and the reasons for this remain unclear. Multiple factors must be considered simultaneously, as the hydrophobicity of PEG increases along with the loosening of PIC core as ionic strength rises. Additionally, the presence of silica gel nanoparticles may further complicate the mechanism, and these combined complexities could obscure clear distinctions in the system.

The gelation tendency of the composite of Nano^Lys/PSS^ with Snowtex® 30 was nearly identical to that of Snowtex® XS, with both of them having relatively small particle sizes (4–6 nm for Snowtex® XS and 10–15 nm for Snowtex® 30) (Figure 5c,d). In contrast, larger Snowtex® nanoparticles (40–60 nm for Snowtex® XL and 70–100 nm for Snowtex® ZL) exhibited different behavior. Although they formed aggregates with Nano^Lys/PSS^ without precipitation during DLS measurements (Figure S6, [polymer] = 1.0 mg/mL), they did not induce gelation with increasing temperature. The underlying mechanism remains unclear, but an appropriate combination of silica gel nanoparticles with PIC flower micelles can achieve high mechanical strength suitable for practical applications.

## Conclusion

We have designed and developed poy(_L_-lysine)-based triblock copolymers (PLys-*block*-PEG-*block*-PLys) as a polycation to prepare flower micelles with polyanions, which convert to hydrogel with increasing temperature. PLys-*block*-PEG-*block*-PLys formed polyion complex (PIC) with PAAc or PSS in water, resulting in the formation of flower micelles (Nano^Lys/PAAc^ or Nano^Lys/PSS^). These flower micelles were around 10–46 nm nanoparticles confirmed by DLS, and ^1^H NMR spectra indicated that PEG formed the hydrophilic shells and PIC formed the micelle-core in water. The PIC flower micelles also exhibited irreversible hydrogel formation even at a low concentration (~40 mg/mL). This sol-gel transition of Nano^Lys/PSS^ was a dual-responsive phenomenon, which needed temperature and ionic strength. Notably, gelation temperature decreased with increasing NaCl concentration. To form stable flower micelles and achieve temperature-triggered gel formation, the PLys chain requires 20 or more repeating Lys units. Nano^Lys/PAAc^ also formed hydrogels, but the gelation temperature was independent of NaCl concentration, suggesting that this temperature-triggered gelation is primarily driven by micelle aggregation due to the hydrophobization of PEG chains, rather than by core loosening caused by changes in ionic strength. The incorporation of silica gel nanoparticles, ranging from a few nanometers to around 15 nm in size, into Nano^Lys/PSS^ enabled us to enhance the mechanical strength of the gel formed upon heating. The gelation ability and mechanical strength of this composite greatly depend on the size of silica particles. For example, when silica particles larger than 40 nm were incorporated, gelation did not occur. In contrast, the incorporation of smaller silica gel nanoparticles induced gelation upon heating, and the mechanical strength of the resulting gel improved dramatically compared to the case without silica. In fact, when 4–6 nm-sized Snowtex® XS was incorporated to Nano^Lys/PSS^, the storage modulus of the resulting hydrogel exceeded 10 kPa, while the gelation temperature remained nearly unchanged, providing sufficient mechanical strength for practical applications. Namely, we achieved a nearly 10-fold increase in mechanical strength compared to our previous PIC flower micelles by utilizing a one-liquid system incorporating silica gel nanoparticles. These fundamental characterizations of irreversible hydrogel formation will contribute to the development of injectable hydrogels capable of continuously delivering Lys, as well as other ionic amino acids and medical drugs, using silica gel composites. Several aspects of this work remain unresolved, requiring further investigation. Specifically, we need to examine the impact of the preparation process on hydrogel formation and properties, the inner structure of composite nanoparticles formed by flower micelles with silica gel nanoparticles, and biocompatibility and cytotoxicity of this system. The potential advantages of our injectable hydrogels for in vivo will be explored separately in future work.

## Supplementary Material

Supplemental Material

## Abbreviations

PLys: _L_-Lysine
PEG: poly(ethylene glycol)
NCA-Lys(Z): *α*-amino acid *N*-carboxyanhydride of *N*^ε^-Carbobenzoxy-_L_-lysine
PLys(Z)-*block*-PEG-*block*-PLys(Z): poly(*N*^ε^-carbobenzoxy-_L_-lysine)-*block*-poly(ethylene glycol)-*block*-poly(*N*^ε^-carbobenzoxy-_L_-lysine)
PLys-*block*-PEG-*block*-PLys: poly(_L_-lysine)-*block*-poly(ethylene glycol)-*block*-poly(_L_-lysine)
PAAc: poly(acrylic acid)
AAc: acrylic acid
PSS: poly(styrenesulfonate)
SS: styrenesulfonate
DP: degree of polymerization
PIC: polyion complex
Nano^Lys/PAAc^: flower micelle-type nanoparticles composed of PLys-*block*-PEG-*block*-PLys and PAAc
Nano^Lys/PSS^: flower micelle-type nanoparticles composed of PLys-*block*-PEG-*block*-PLys and PSS
Nano^Lys/PSS/SiO^: flower micelle-type nanoparticles composed of PLys-*block*-PEG-*block*-PLys, PSS, and silica gel nanoparticles
ST-XS: Snowtex® XS
ST-30: Snowtex® 30
ST-XL: Snowtex® XL
ST-ZL: Snowtex® ZL
Nano^Lys/PSS/SiO^(**P4**;ST-XS): flower micelle-type nanoparticles composed of PLys-*block*-PEG-*block*-PLys(**P4**), PSS, and Snowtex® XS
GPC: gel permeation chromatography
NMR: nuclear magnetic resonance
DLS: dynamic light scattering
TEM: transmission electron microscope
*D*_H,volume_: volume distribution of hydrodynamic diameter
*G*’: storage modulus
*G*: oss modulus
DMF: *NN*-dimethylformamide
TFA: trifluoroacetic acid
DMSO: dimethyl sulfoxide
PB solution: phosphate-buffered solution
