# Supplementary material for: Poly(L-lysine)-block-poly(ethylene glycol)-block-poly(L-lysine) triblock copolymers for the preparation of flower micelles and their irreversible hydrogel formation
Source: Sci Technol Adv Mater. 2024 Nov 25;26(1):2432856. doi: 10.1080/14686996.2024.2432856 (PMC11703508; doi:10.1080/14686996.2024.2432856)
Supplement: Supplemental Material [file TSTA_A_2432856_SM1116.pdf]

Supplementary Information

Poly(L-lysine)-*block*-poly(ethylene glycol)-*block*-poly(L-lysine) triblock copolymers for the preparation of flower micelles and their irreversible hydrogel formation

Yuta Koda[a,*], and Yukio Nagasaki[a,b,c,d,e]

a) Department of Materials Science, Institute of Pure and Applied Sciences, University of Tsukuba, 1-1-1 Tennoudai, Tsukuba, Ibaraki, 305–8573, Japan
b) Master's School of Medical Sciences, Graduate School of Comprehensive Human Sciences, University of Tsukuba, 1-1-1 Tennoudai, Tsukuba, Ibaraki, 305- 8573, Japan
c) Center for Research in Radiation, Isotope and Earth System Sciences (CRiES), University of Tsukuba, 1-1-1 Tennoudai, Tsukuba, Ibaraki, 305-8573, Japan
d) Department of Chemistry, Graduate School of Science, The University of Tokyo, Bunkyo-ku, Tokyo, 113-0033, Japan
e) High-value Biomaterials Research and Commercialization Center (HBRCC), National Taipei University of Technology, Taipei, 10608, Taiwan

Email: koda@ims.tsukuba.ac.jp
*: corresponding author

ORCID
Yuta Koda: 0000-0003-1724-2359
Yukio Nagasaki: 0000-0001-7975-6510

Table of Contents

Supporting Data S3

Figure S1. GPC curves of **P1**, **P2**, and **P3** S3

Figure S2. Volume distribution of $D_{H,volume}$ of Nano$^{Lys/PAAc}$ S4

Figure S3. Volume distribution of $D_{H,volume}$ of Nano$^{Lys/PSS}$ S4

Figure S4. A TEM image and ^{1}H NMR analysis of Nano$^{Lys/PSS}$(**P2**) S5

Figure S5. Volume distribution of $D_{H,volume}$ of Nano$^{Lys/PSS/SiO}$(**P4**;ST-XS) S6

Figure S6. Volume distribution of $D_{H,volume}$ of Nano$^{Lys/PSS/SiO}$(**P4**) S7

Figure S7. Schematic illustration of Nano$^{Lys/PSS/SiO}$(**P4**) S7

Table S1. Gelation Points of PLys-Based Block Copolymers with Polyanions S8

Supporting Data

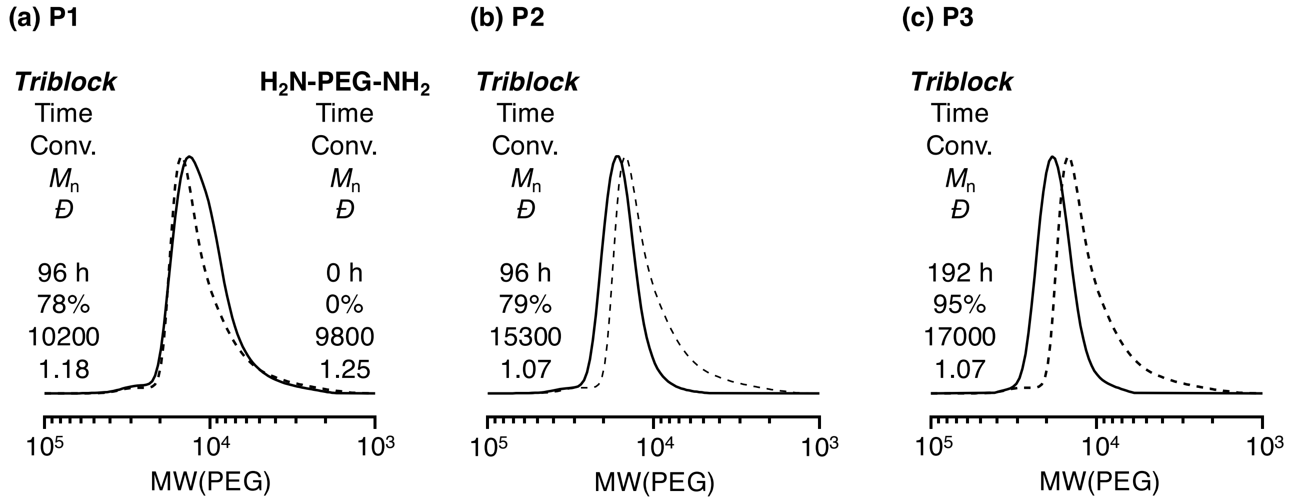

Figure S1. GPC curves of PLys(Z)-*block*-PEG-*block*-PLys(Z)(**P1**–**P3**) obtained by the ring-opening polymerization of NCA-Lys(Z) in DMF at 45°C.

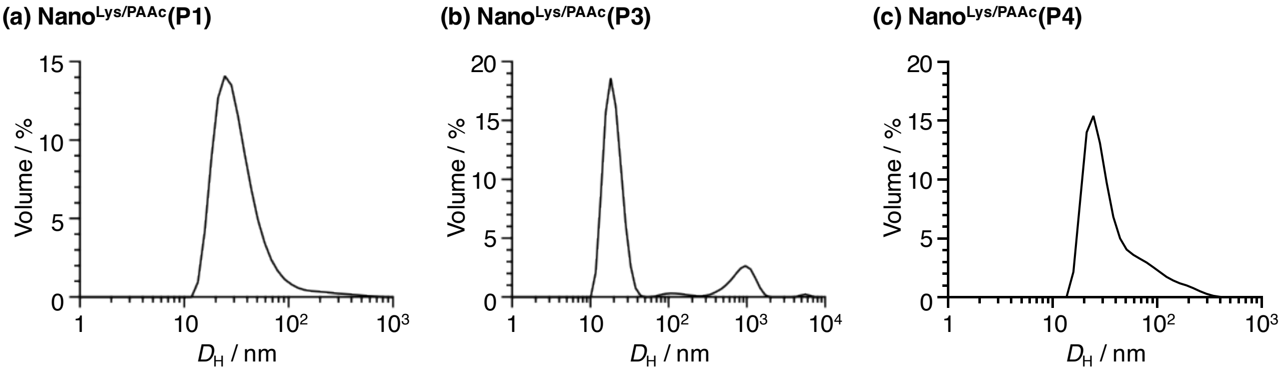

Figure S2. Volume distribution of the hydrodynamic diameter of Nano$^{Lys/PAAc}$(a, **P1**; b, **P3**; c, **P4**) in water ([polymer] = 10 mg/mL, Lys unit / AAc unit = 1/1 (mol/mol)).

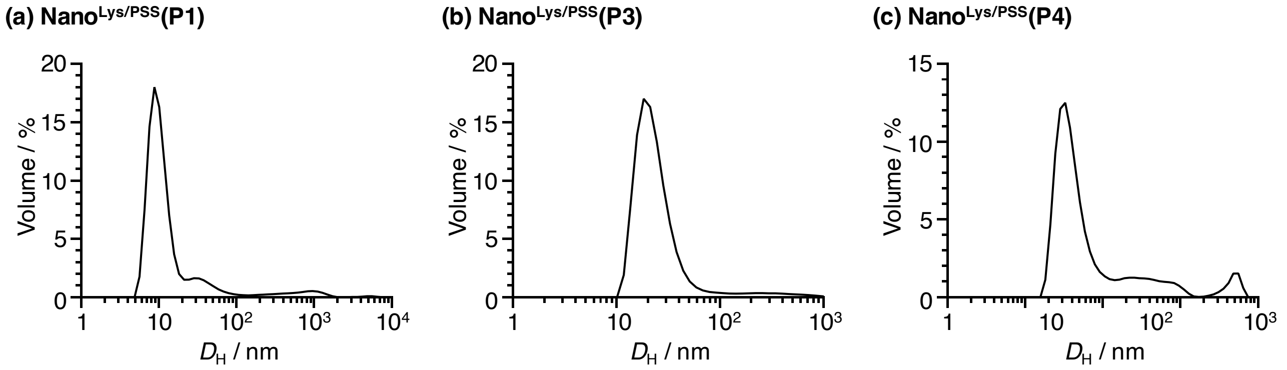

Figure S3. Volume distribution of the hydrodynamic diameter of Nano$^{Lys/PSS}$(a, **P1**; b, **P3**; c, **P4**) in water ([polymer] = 10 mg/mL, Lys unit / styrenesulfonate unit = 1/1 (mol/mol)).

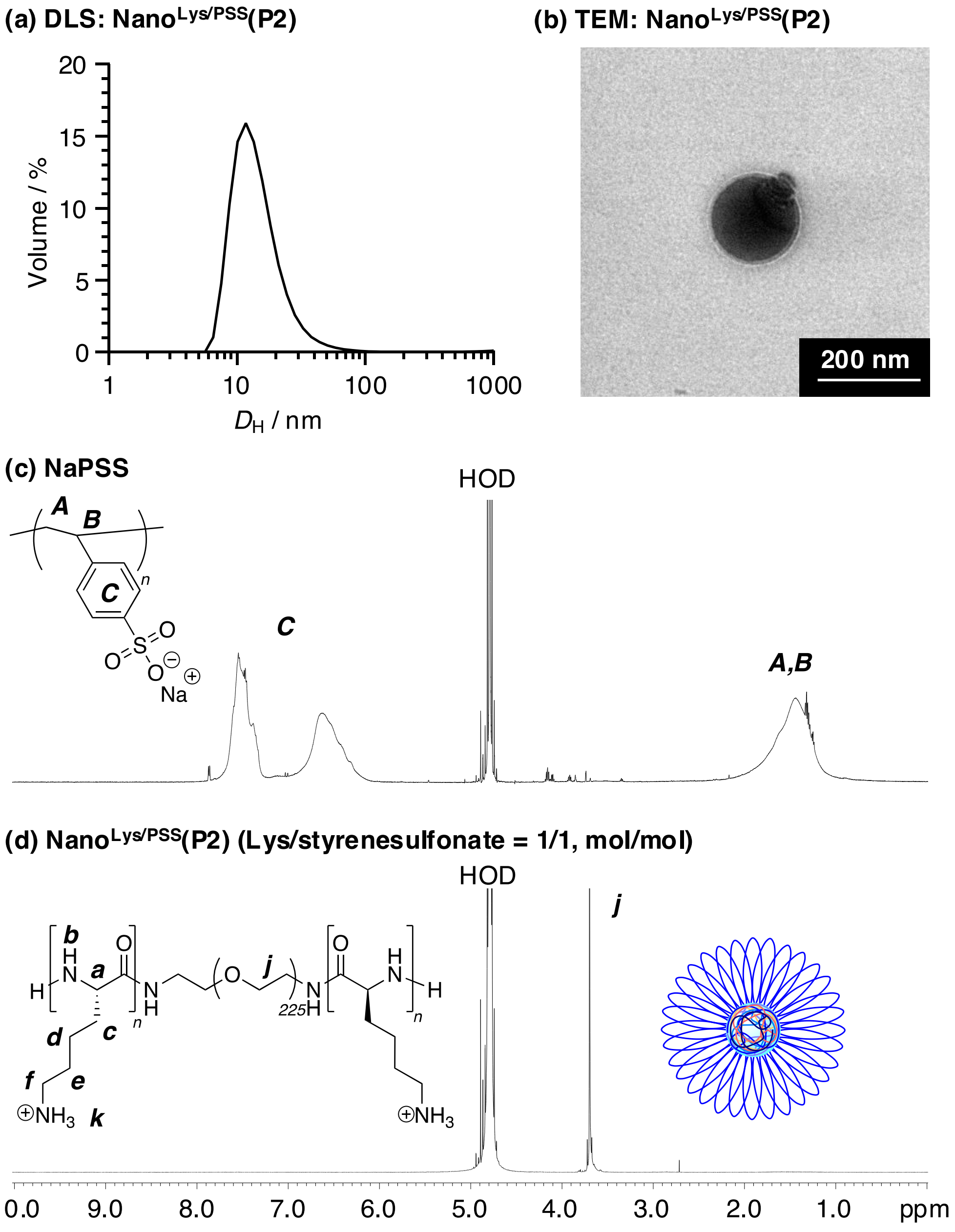

Figure S4. (a) Volume distribution of the hydrodynamic diameter and (b) a TEM image of PIC flower micelles (Nano$^{Lys/PSS}$(**P2**)) in water at 25 °C ([polymer] = 10 mg/mL, Lys unit in PLys-*block*-PEG-*block*-PLys(**P2**) / styrenesulfonate unit in PSS = 1 / 1 (mol/mol)). ^{1}H NMR spectra (600 MHz) of (c) NaPSS and (d) Nano$^{Lys/PSS}$(**P2**) ([polymer] = 5.0 mg/mL, δ = 4.79 ppm (HOD)).

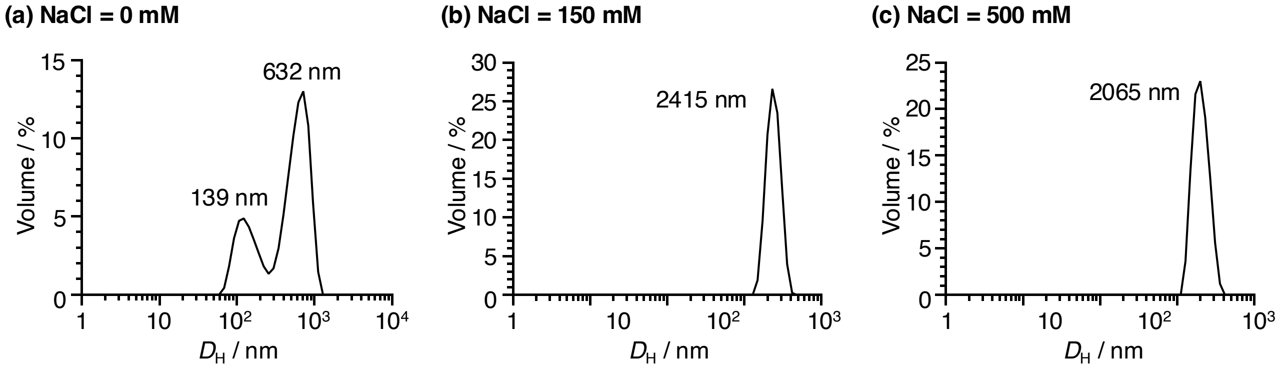

Figure S5. Volume distribution of the hydrodynamic diameter of Nano$^{Lys/PSS}$(**P4**;ST-XS) in water ([polymer] = 1.0 mg/mL, Lys unit in PLys-*block*-PEG-*block*-PLys(**P4**) / styrenesulfonate unit in PSS = 1/1 (mol/mol), [Snowtex® XS (ST-XS)] = 56 mg/ML; [NaCl] = (a) 0, (b) 150, and (c) 500 mM).

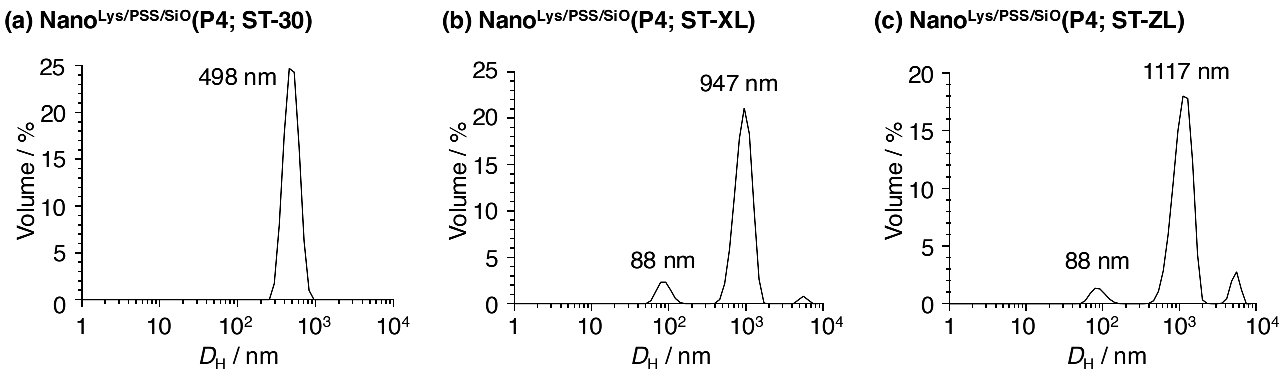

Figure S6. Volume distribution of the hydrodynamic diameter of $Nano^{Lys/PSS/SiO}$(**P4**; a, Snowtex® 30 (ST-30); b, Snowtex® XL (ST-XL); c, Snowtex® ZL (ST-ZL)) in water ([polymer] = 1.0 mg/mL, Lys unit in PLys-*block*-PEG-*block*-PLys(**P4**) / styrenesulfonate unit in PSS = 1/1 (mol/mol), [Snowtex®] = 56 mg/mL; [NaCl] = 0 mM).

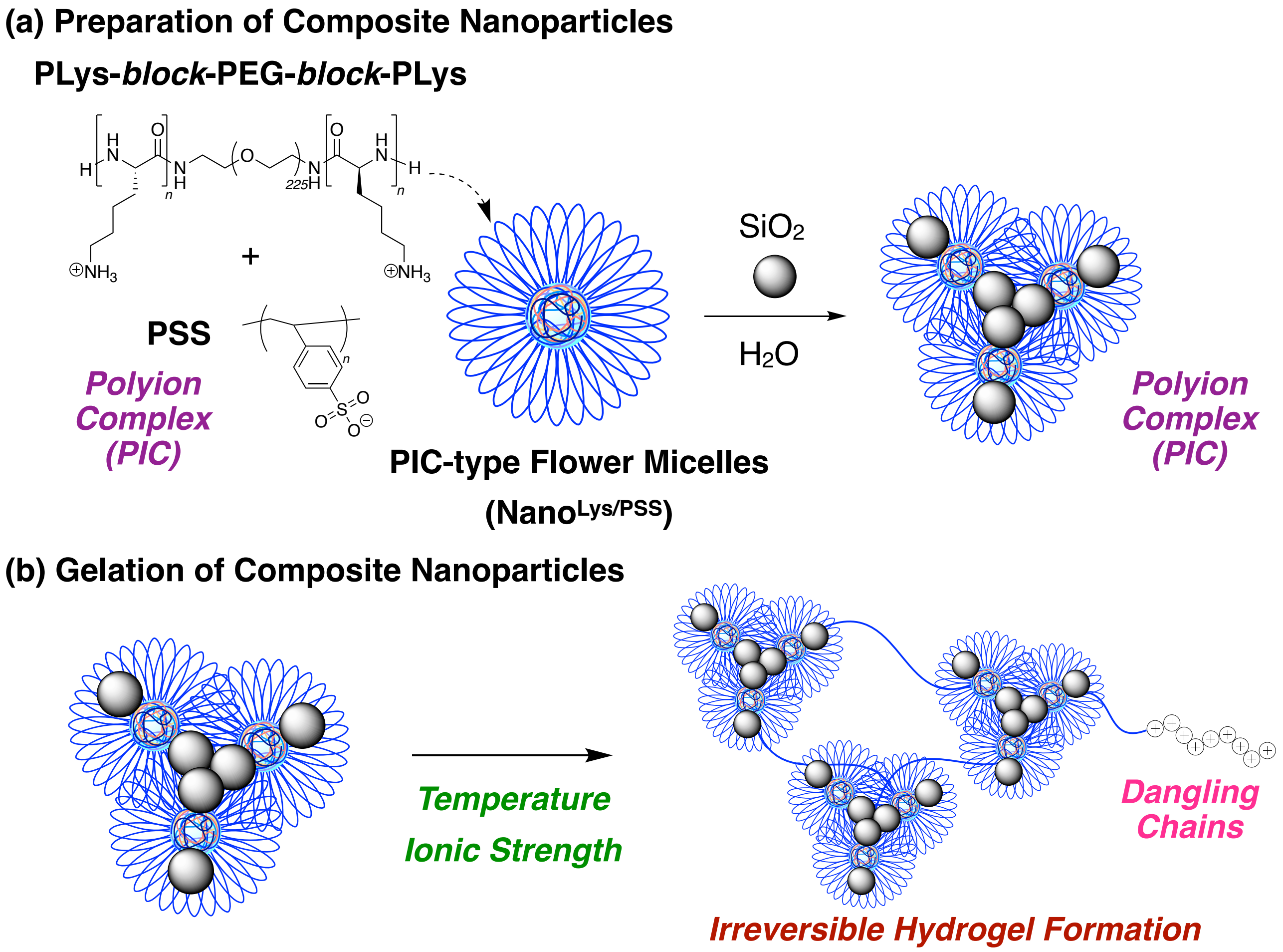

Figure S7. Schematic illustration of irreversible hydrogel formation of $Nano^{Lys/PSS/SiO}$(**P4**).

Table S1. Gelation Points of PLys-Based Triblock Copolymers with Polyanions

Entry	Polycations	Polyanions	Silica	NaCl / mM	T_{gel} [a]/ °C
1	**P1**			150	None
2	**P2**	NaPSS	None	150	None
3	**P3**			150	n.d.
4				0	–
5	**P4**	NaPSS	None	150	25.9
6				500	24.7
7				0	26.5
8	**P4**	PAAc	None	150	26.5
9				500	27.0
10				0	26.5
11	**P4**	NaPSS	ST-XS	150	30.1
12				500	31.3
13			ST-30	150	–
14	**P4**	NaPSS	ST-XL	150	25.9
15			ST-ZL	150	25.9

[a] Temperature of gelation points (T_{gel}) was determined at the cross-point of *G'* and *G"* in the heating process.
